# An epithelial-mesenchymal transition-related 5-gene signature predicting the prognosis of hepatocellular carcinoma patients

**DOI:** 10.1186/s12935-021-01864-5

**Published:** 2021-03-12

**Authors:** Gongmin Zhu, Hongwei Xia, Qiulin Tang, Feng Bi

**Affiliations:** Department of Abdominal Oncology, Cancer Center and Laboratory of Molecular Targeted Therapy in Oncology, West China Hospital, Sichuan University, No.37 guoxue lane, Chengdu, 610041 Sichuan Province China

**Keywords:** Epithelial-mesenchymal transition, Hepatocellular carcinoma, Prognostic signature, PDCD6, TCGA

## Abstract

**Background:**

Tumor metastasis is one of the leading reasons of the dismal prognosis of hepatocellular carcinoma (HCC). Epithelial-mesenchymal transition (EMT) is closely associated with tumor metastasis including HCC. The purpose of this study is to construct and validate an EMT-related gene signature for predicting the prognosis of HCC patients.

**Methods:**

Gene expression data of HCC patients was downloaded from The Cancer Genome Atlas (TCGA) database. Gene set enrichment analysis (GSEA) was performed to found the EMT-related gene sets which were obviously distinct between normal samples and paired HCC samples. Cox regression analysis was used to develop an EMT-related prognostic signature, and the performance of the signature was evaluated by Kaplan–Meier curves and time-dependent receiver operating characteristic (ROC) curves. A nomogram incorporating the independent predictors was established. Quantitative real-time polymerase chain reaction (qRT-PCR) was used to detect the expression levels of the hub genes in HCC cell lines, and the role of PDCD6 in the metastasis of HCC was determined by functional experiments.

**Results:**

An EMT-related 5-gene signature (PDCD6, TCOF1, TRIM28, EZH2 and FAM83D) was constructed using univariate and multivariate Cox regression analysis. Based on the signature, the HCC patients were classified into high- and low-risk groups, and patients in high-risk group had a poor prognosis. Time-dependent ROC and Cox regression analyses suggested that the signature could predict HCC prognosis exactly and independently. The predictive capacity of the signature was also validated in two external cohorts. GSEA results showed that many cancer-related signaling pathways such as PI3K/Akt/mTOR pathway and TGF-β/SMAD pathway were enriched in high-risk group. The result of qRT-PCR revealed that PDCD6, TCOF1 and FAM83D were highly expressed in HCC cancer cells. Among them, PDCD6 were found to promote cell migration and invasion.

**Conclusion:**

The EMT-related 5-gene signature can serve as a promising prognostic biomarker for HCC patients and may provide a novel mechanism of HCC metastasis.

**Supplementary Information:**

The online version contains supplementary material available at 10.1186/s12935-021-01864-5.

## Background

Liver cancer ranks sixth in aspect of new cases and becomes the fourth main cause of cancer-related death worldwide in 2018 [1]. Hepatocellular carcinoma (HCC), the most prevalent type of primary liver cancer, typically develops in the setting of cirrhosis, with chronic hepatitis B, chronic hepatitis C, alcohol abuse and non-alcoholic fatty liver disease [2]. Although many advanced management strategies have presented considerable effects on diagnosis of HCC, the overall survival (OS) of patients with HCC remains poor on account of the high metastasis rate [3]. Besides, approximately 70% of HCC patients receiving surgical resection or ablation have tumor recurrence within 5 years, which severely affects the prognosis of patients [4]. Therefore, identification of novel prognostic biomarkers is urgently needed for predicting clinical outcome of HCC patients and providing decision for surgery, pharmacological intervention and conservative treatments.

Epithelial-mesenchymal transition (EMT) is a process that epithelial cells lose polarized organization and obtain the abilities of migratory and invasive [5]. About 80% of human malignancies originate from epithelial tissues including lung, breast, colon, liver, etc. [6]. Neoplastic cells of these tumors at early stage retain the characteristics of epithelial cells such as lacking motility and failing to form continuous cell sheets. However, in advanced carcinoma, tumor cells present mesenchymal properties with highly aggressive, which are associated with metastatic dissemination [7, 8]. Moreover, these mesenchymal features can reverse through mesenchymal-epithelial transition process, which make tumor cells restore the epithelial phenotype and enable them relocate into the metastasis sites [9]. Besides, EMT can also play a pivotal role in increasing the frequency of cancer stem cells which have capability to self-renew and produce more differentiated derivatives [10]. Therefore, EMT programs are closely associated with the development and progression of various malignancies including hepatocellular carcinoma. Recent study has reported that Thioredoxin domain-containing protein 12 promotes EMT through interaction with β-catenin and transcriptional activation of ZEB1, which contributes to HCC metastasis and indicates an adverse clinical outcome [11]. Currently, with the easy accessibility of many public databases, accumulating signatures or biomarkers has been developed to predict the prognosis of patients, whereas the EMT-related prognostic model for HCC patients is still lacking.

In the present study, we constructed an EMT-related 5-gene signature using The Cancer Genome Atlas (TCGA) database to predict the prognosis of patients with HCC. The predictive effectiveness and accuracy of the signature was validated using the International Cancer Genome Consortium (ICGC) database and one microarray dataset (GSE76427) from the Gene Expression Omnibus (GEO). Next, based on the 5-gene signature and American Joint Committee on Cancer (AJCC) stage, we established a nomogram to evaluate clinical significance. Then, through gene set enrichment analysis (GSEA), we found that HCC patients with high risk score are more liable to progression and metastasis, which is in line with the characteristics of EMT. Finally, we detected the expression of the five genes and further explored the function of PDCD6 in HCC cells, and found that PDCD6 played a vital role in HCC metastasis.

## Materials and methods

### Data acquisition

Raw gene expression data of HCC (n = 374) and normal samples (n = 50), and clinical information of the corresponding patients were obtained from the TCGA data portal (http://portal.gdc.cancer.gov/projects). Stemness scores based on mRNA (RNAss) of TCGA pan-cancer data was downloaded from xena browser (https://xenabrowser.net/datapages/). For clinical information, samples with any missing data or a follow-up time less than one month were excluded, and finally a total of 319 HCC samples were applied for subsequent study.

For two validation cohorts, LIRI-JP cohort was downloaded from ICGC portal (http://dcc.icgc.org/release/current/Projects) and a microarray dataset (GSE76427) was downloaded from the GEO database (https://www.ncbi.nlm.nih.gov/geo/). According to the same screening criteria of TCGA cohort, a total of 232 samples from ICGC cohort and 94 samples from GSE76427 were selected for external validation. Detailed information of TCGA cohort and two validation cohorts were shown in Table 1. Due to all data were downloaded from the public database, it was not needed to acquire additional ethical approval for this study.

**Table 1:** Clinical information of patients with hepatocellular carcinoma in three datasets

Characteristics	TCGA (N = 319)	ICGC (N = 232)	GSE76427 (N = 94)
Age (years) (%)			
≤ 60	160 (50.2)	49 (21.1)	43 (45.7)
> 60	159 (49.8)	183 (78.9)	51 (54.3)
Gender (%)			
Female	100 (31.3)	61 (26.3)	12 (12.8)
Male	219 (68.7)	171 (73.7)	82 (87.2)
Grade (%)			
G1	44 (13.8)	NA	NA
G2	154 (48.3)	NA	NA
G3	109 (34.1)	NA	NA
G4	12 (3.8)	NA	NA
AJCC stage (%)			
Stage I	160 (50.2)	36 (15.5)	41 (43.6)
Stage II	76 (23.8)	104 (44.8)	31 (33.0)
Stage III	80 (25.1)	73 (31.5)	19 (20.2)
Stage IV	3 (0.9)	19 (8.2)	3 (3.2)
Survival status (%)			
Alive	215 (67.4)	189 (81.5)	74 (78.7)
Deceased	104 (32.6)	43 (18.5)	20 (21.3)

### Gene set enrichment analysis (GSEA)

To identify EMT-related gene sets in 50 normal samples and paired HCC samples from TCGA cohort, analysis was performed using GSEA software 4.1.0 from the Broad Institute [12]. The Hallmark gene sets “h.all.v7.2.symbols.gmt”, GO gene sets “c5.bp.v7.2.symbols.gmt”, KEGG gene sets “c2.cp.kegg.v7.2.symbols.gmt”, BioCarta gene sets “c2.cp.biocarta.v7.2.symbols.gmt”, PID gene sets “c2.cp.pid.v7.2.symbols.gmt”, and Reactome gene sets “c2.cp.reactome.v7.2.symbols.gmt” were downloaded from Molecular Signatures Database (http://software.broadinstitute.org/gsea/msigdb/index.jsp). Permutations were performed 1000 times to analyze each gene set and acquire a normalized enrichment score (NES). The gene set with normalized P-value < 0.05 and false discovery rate (FDR) < 0.25 was chosen for subsequent analysis. Finally, 152 EMT-related genes from GO_EPITHELIAL_TO_MESENCHYMAL_TRANSITION gene set were considered as core genes.

### Construction and validation of the prognostic EMT-related gene signature

The 152 EMT-related genes of the 50 normal samples and paired tumor were comparatively analyzed to identify the significantly up-regulated genes in tumor samples (log2 fold change > 1 and FDR < 0.05). The 319-patient TCGA cohort was used as discovery cohort to construct a prognostic model. Univariate Cox regression analysis was conduct to identify up-regulated genes that were related to survival, and genes with P-value < 0.05 were used for subsequent multivariate Cox regression analysis. Finally, an EMT-related 5-gene signature was developed for prognosis prediction, and the risk score of each patient was calculated as the following formula:

$$ {\text{Risk score}} = \sum\limits_{{{\text{i}} = 1}}^{{\text{n}}} {\left( {{\text{Coef}}_{{\text{i}}} \times {\text{Exp}}_{{\text{i}}} } \right)} $$

Where n is a number of prognostic genes, and Coef_i_ indicates the estimated regression coefficient of the gene, and Exp_i_ indicates the expression level of the gene. Patients were divided into high-risk or low-risk group based on the medium value of risk score.

To verify the predictive capacity of 5-gene signature, the 232 HCC patients from the ICGC database and 94 HCC patients from the GSE76427 were served as validation cohorts. With the medium score as the cut-off value, patients were also classified into high- or low-risk group for survival analysis.

### Development and assessment of a predictive nomogram

The nomogram containing independent prognostic factors was plotted utlizing the “rms” package in R software. The total score of each patient could be calculated using the nomogram, which could be applied to predict the 1-, 3- and 5-year survival rate of HCC patients. Calibration curves were utilized to evaluate the performance of the nomogram. In the calibration graph, nomogram-predicted survival and the observed clinical outcome are plotted on the x-axis and y-axis, respectively, and the 45° dotted line represents the optimal prediction.

### Bioinformatics analysis

To identify the regulatory network of the 5 EMT-related genes, analysis was conducted using GeneMANIA (http://www.genemania.org/) [13]. The cBio Cancr Genomics Portal (cBioPortal) (http://cbioportal.org), an open-access resource for visualization and exploration of various cancer genomics data [14], was performed to analyze genetic alterations of prognostic genes in HCC patients from TCGA cohort.

### Cell culture

Human HCC cell lines SK-Hep-1, HepG2, Hep3B and human normal liver cell line HL-7702 were grown in Dulbecco’s modified Eagle’s medium (Gibco, Grand Island, NY, USA) supplemented with 10% fetal bovine serum (FBS, Gibco, USA) and 1% penicillin–streptomycin in a 37 °C incubator containing 5% CO_2_.

### Small interfering RNA interference assay

Small interfering RNA (siRNA) was designed and purchased from RIBOBIO (Guangzhou Ribobio Co., Ltd., Guangzhou, China). The sequences of siRNAs were as follow: si-PDCD6-1 (5′-GTAGCTGTATCGTTCTAAT-3′) and si-PDCD6-2 (5′-GCAGTTAGATGCTGTTCTT-3′). Additionally, a scrambled siRNA was synthesized as negative control (NC). SiRNA transfections were performed using Lipofectamine 2000 (Invitrogen, Carlsbad, CA, USA) according to the protocol of manufacturer.

### RNA extraction and quantitative real-time polymerase chain reaction (qRT-PCR)

Total RNA was extracted from cell lines using Cell Total RNA Isolation kit (Foregene, Chengdu, China) following the manufacturer’s instruction. RNA (1 μg) was reverse‐transcribed to complementary DNA (cDNA) according to the protocol of the PrimeScript RT reagent kit (TaKaRa, Osaka, Japan). qRT-PCR was carried out on the iQ5 Real-Time PCR Detection System (Bio-Rad, Hercules, CA, USA) using the SYBR Green qPCR Supermixes (Bio-Rad). Primer sequences were listed in Table 2. Cycle threshold (Ct) of each gene was standardized to GAPDH using the 2^−ΔΔCT^ method. Each experiment was conducted in triplicate.

**Table 2:** Primer sequences for five hub genes and β-actin

Gene	Primer sequence
PDCD6	Forward: ATGGCCGCCTACTCTTACC
	Reverse: TCCTGTCTTTATCGACCCTCTG
TCOF1	Forward: AGTCTCCAGCAAGAAAGGCG
	Reverse: TCCTCTTCTGGCTTCTTGGC
TRIM28	Forward: TGTTTCCACCTGGACTGTCA
	Reverse: CCAGCAGTACACGCTCACAT
EZH2	Forward: TGCAGTTGCTTCAGTACCCATAAT
	Reverse: ATCCCCGTGTACTTTCCCATCATAAT
FAM83D	Forward: AGAGCGGCAATTCCACTTCG
	Reverse: TGCCAGAATGAAGGCCAAGG
β-actin	Forward: CATGTACGTTGCTATCCAGGC
	Reverse: CTCCTTAATGTCACGCACGAT

### Western blot analysis

RIPA buffer (Beyotime, Haimen, China) supplemented with protease inhibitors (Roche) was used for cell lysis. A BCA protein assay kit (Beyotime) was used for protein concentration detection. Total protein (30 μg) was separated in a 10% sodium dodecyl sulfate‐polyacrylamide gel and transferred to polyvinylidene fluoride membranes (0.45 μm, Millipore, Bedford, MA, USA). After blocking with 5% skim milk, the membranes were incubated with primary anti-PDCD6 (1:2000; 12,303–1-AP, Proteintech, Rosemont, IL) and anti-GAPDH (1:1000; Santa Cruz, USA) at 4 °C overnight. After a washing step, membranes were incubated with horseradish peroxidase‐conjugated goat anti‐rabbit or anti-mouse secondary antibodies (1:3000). Later, the immunoreactive bands were detected using enhanced chemiluminescence system.

### Wound healing assay

Cells were planted into 6-well plates for 24 h to reach 80–90% confluence. Lines were scratched across cells with a sterile pipette tip. The cells were washed by phosphate buffer saline (PBS) and were cultured in serum-free medium. After 0, 24 and 48 h of wounding, images were captured to measure the area of cell migration. The relative wound closure = (the gap area of the wound at 0 h − the gap area of the wound at 24 h or 48 h in si-PDCD6 group)/(the gap area of the wound at 0 h − the gap area of the wound at 24 h or 48 h in NC group). The experiment was conducted in triplicate.

### Transwell invasion assay

The transwell chambers (8 μm pore size, Corning, NY, USA) coated with Matrigel (BD Biosciences, NJ, USA) were placed into a 24-well plate. A total of 1 × 10^5^ cells/well were seeded into the upper chamber containing 200 μl of serum-free medium, whereas 600 μl medium supplemented with 10% FBS was added to the lower chamber. Following incubation for 36–48 h (HepG2, 36 h; Hep3B, 48 h) at 37 °C incubator containing 5% CO_2,_ cells inside the chamber were wiped off. Then, cells on the bottom of the membrane were fixed in 4% paraformaldehyde and stained with 0.1% crystal violet. Images of 3 random fields in each chamber were captured under a microscope and the number of cells was counted.

### Statistical analysis

Statistical analyses were performed with Rstudio (version 1.3.1093), SPSS 22.0 software (Armonk, NY), and GraphPad Prism 5.0 software (San Diego, CA, USA). Kaplan–Meier survival curve combined with log-rank test was employed to evaluate survival differences between high- and low-risk groups. Univariate and multivariate Cox proportional hazard regression model were conducted to analyze the hazard ratio of prognostic factors and to identify independent prognostic factors. The predictive accuracy of the prognostic model was reflected by time-dependent receiver operating characteristic (ROC) curve and determined by area under curve (AUC). Associations between the EMT-related gene signature risk level and clinicopathological characteristics were analyzed by chi‐square test. Comparisons between two groups were performed using two tailed Student’s t test or paired t test. Correlations among genes in EMT-related signature were determined by the Spearman rank correlation test. All results are presented as the mean ± standard deviation (SD). A P < 0.05 was considered statistically significant.

## Results

### Identification of EMT-related genes in HCC

**Fig. 1:**
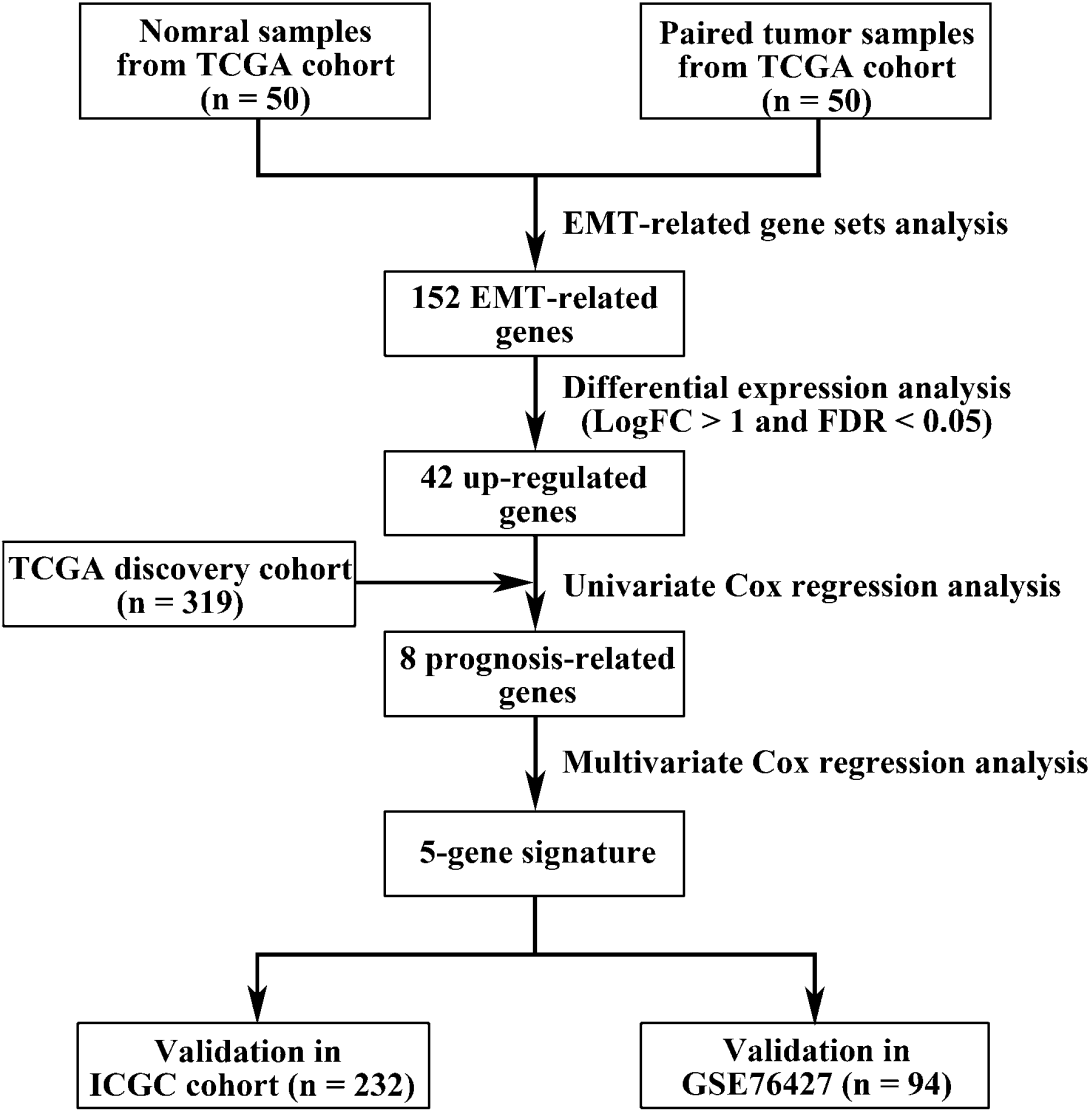
A flow chart detailing the development and validation of the EMT-related 5-gene signature

**Fig. 2:**
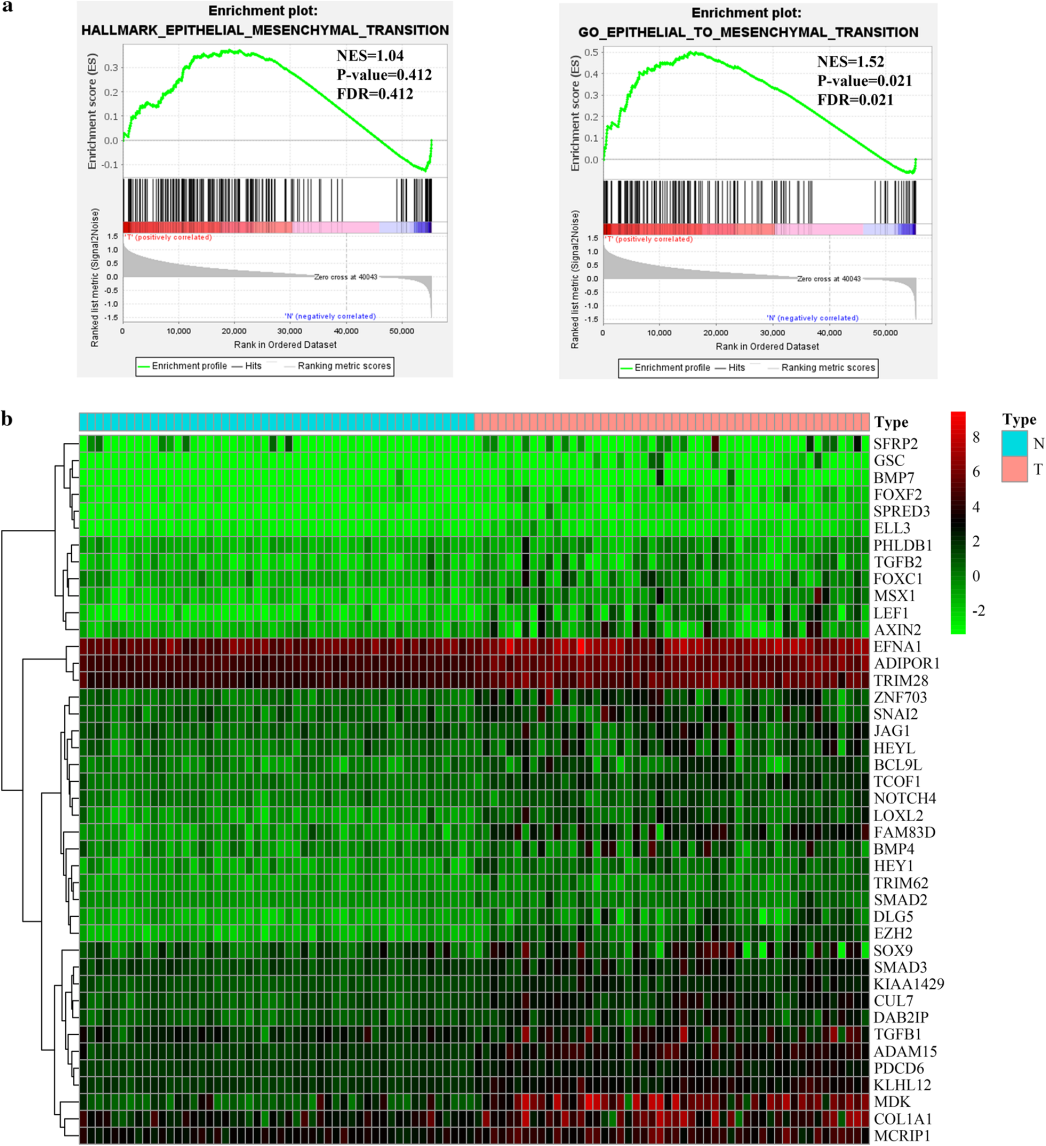
Identification of EMT-related gene sets. **a** GSEA plots of two EMT-related gene sets between normal samples and paired HCC samples. **b** Heatmap of 42 significantly upregulated genes in HCC samples from GO_EPITHELIAL_TO_MESENCHYMAL_TRANSITION gene set

A detailed flow chart illustrating the procedure of our study is depicted in Fig. 1. GSEA was utilized to identify whether two EMT-related gene sets were strikingly distinct between normal samples and paired HCC samples. As the result shown, only GO_EPITHELIAL_TO_MESENCHYMAL_TRANSITION gene set was obviously enriched in HCC samples (Fig. 2a). Then, through differentially expressed gene analysis, we found that 42 EMT-related genes from GO_EPITHELIAL_TO_MESENCHYMAL_TRANSITION gene set were significantly up-regulated in HCC samples, which were selected for further analysis (Fig. 2b).

### Development of the prognostic EMT-related gene signature

By inputting the 42 up-regulated genes identified above into the TCGA discovery cohort, a total of 8 EMT-related genes were shown to be significantly associated with OS using univariate Cox regression analysis. Then, multivariate Cox regression method was utilized for further screening. Finally, 5 genes, PDCD6, TCOF1, TRIM28, EZH2 and FAM83D, were identified as predictive biomarkers of prognosis. A prognostic model based on the 5 EMT-related genes was established to calculate the risk score of each patient as follow: risk score = (0.0495 × expression of PDCD6) + (0.1605 × expression of TCOF1) + (− 0.0070 × expression of TRIM28) + (0.1388 × expression of EZH2) + (0.0485 × expression of FAM83D).

**Fig. 3:**
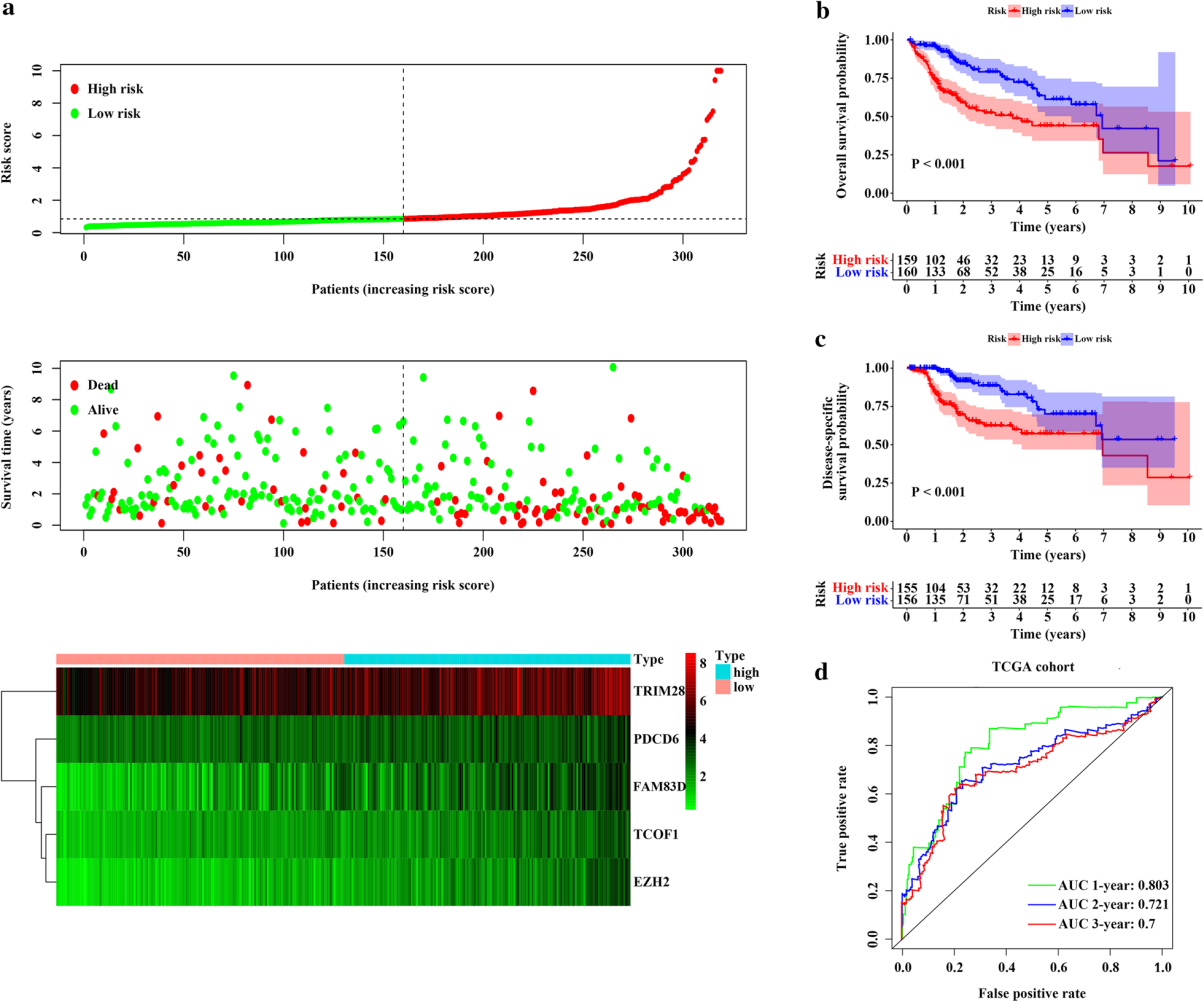
The 5-gene signature predicts the prognosis of HCC patients in TCGA discovery cohort. **a** The distribution of risk score and survival status of each patient. Red indicates high risk or dead, and green indicates low risk or alive. The heatmap of 5 hub genes expression, with red indicating high expression, black indicating moderate expression and green indicating low expression. **b** Kaplan–Meier curve of OS between high- and low-risk groups. **c** Kaplan–Meier curve of DSS between high- and low-risk groups. **d** Time dependent ROC curves for predicting 1-, 2- and 3-year OS

According to the median value of the signature, patients in TCGA cohort were divided into high-risk and low-risk groups. The rise in risk score was paralleled by increases in the expression of the 5 EMT-related genes and in the mortality of HCC patients (Fig. 3a). Likewise, the survival results showed that patients with a high-risk score had worse OS [hazard ratio (HR) = 2.441, 95% confidence interval (CI) 1.654–3.603, P < 0.001] and disease-specific survival (DSS) (HR = 2.823, 95% CI 1.731–4.603, P < 0.001) than those with a low-risk score (Fig. 3b, c). Moreover, the time-dependent ROC curves demonstrated that the AUCs for 1-year, 2-year, and 3-year OS were 0.803, 0.721, and 0.7, which suggested favorable accuracy of the EMT-related signature in predicting the prognosis of HCC patients.

### EMT-related gene signature as an independent indicator in predicting survival

**Fig. 4:**
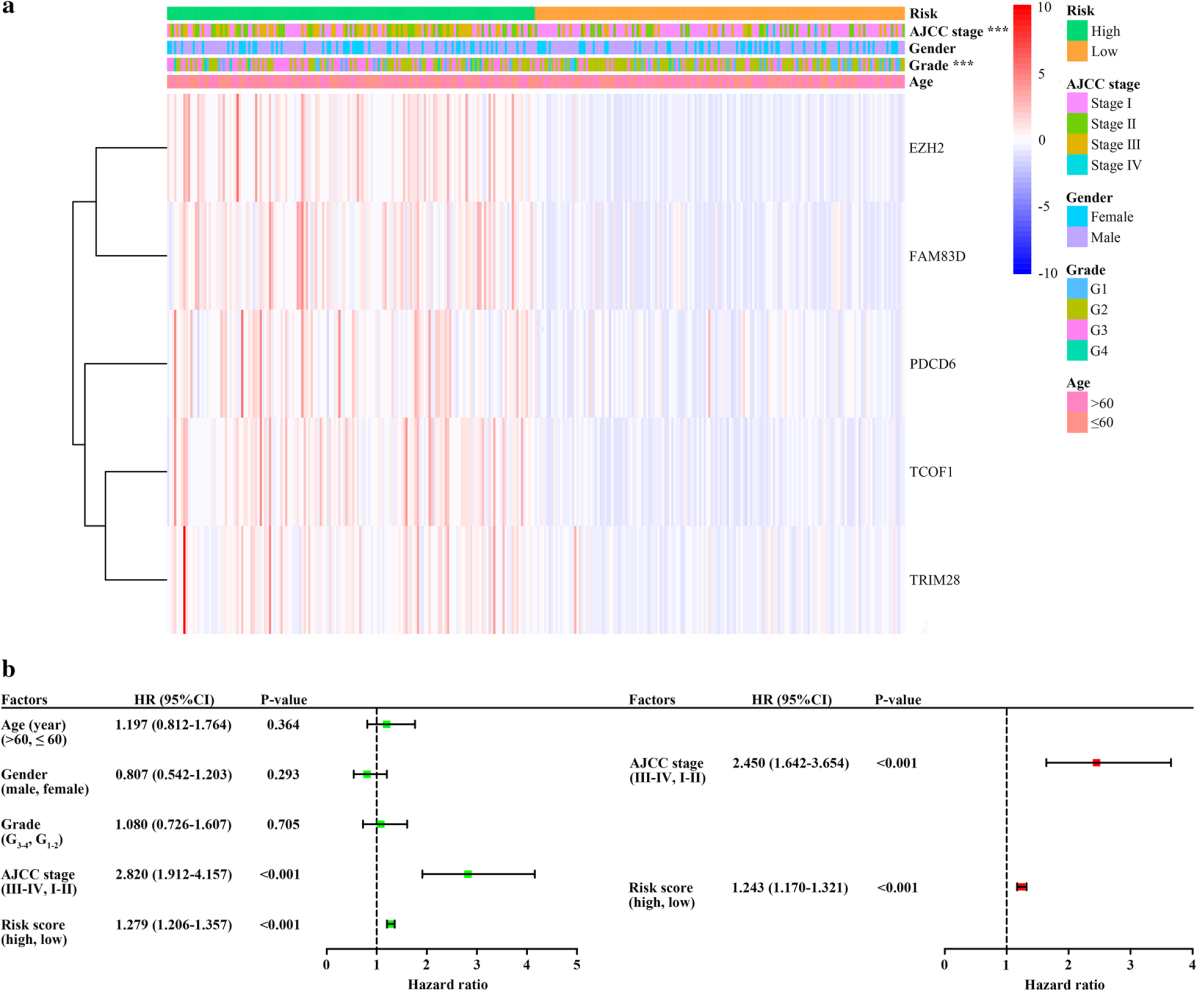
Cox regression analyses for identifying independent prognostic factors. **a** The heatmap demonstrating the association between the 5-gene signature and clinicopathological factors. ***P < 0.001. **b** Univariate and Multivariate Cox regression analyses of prognostic factors

Preliminarily, a cluster heat map of the 5 EMT-related genes was developed to present the association between the signature and clinicopathological characteristics. We found that the risk level was positively related to tumor grade and AJCC stage (Fig. 4a and Additional file 1). To determine whether the EMT-related 5-gene signature could serve as an independent prognostic factor in HCC, we employed univariate and multivariate Cox regression analyses to compare the prognostic value of the signature with several clinicopathological factors including age, gender, tumor grade and AJCC stage. The results indicated that the EMT-related 5-gene signature (HR = 1.243, 95% CI 1.170–1.321, P < 0.001) and AJCC stage (HR = 2.450, 95% CI 1.642–3.654, P < 0.001) were the independent predictors of HCC (Fig. 4b).

### Stratification analysis of the EMT-related gene signature

**Fig. 5:**
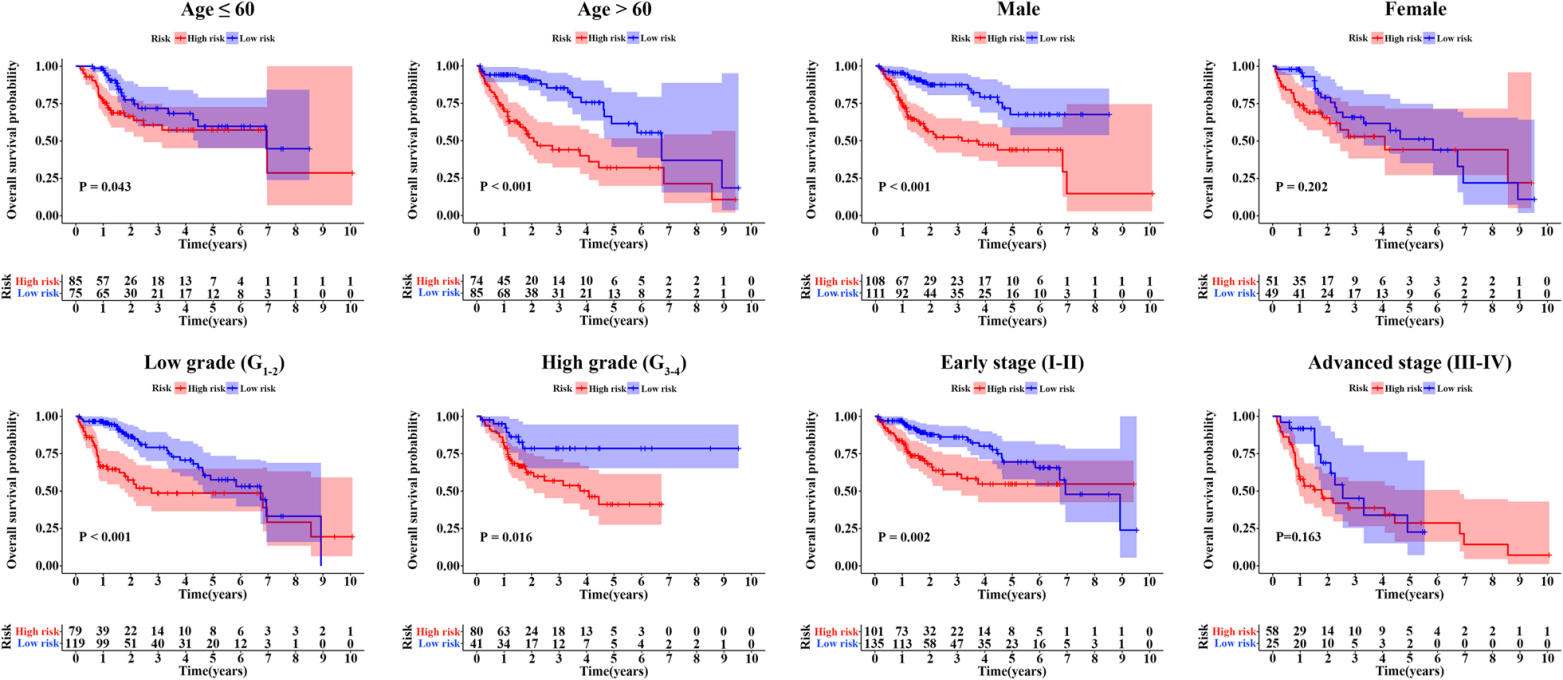
Kaplan–Meier subgroup analysis of HCC patients in TCGA discovery cohort based on the 5-gene signature stratified by clinical characteristics

We conducted stratification analysis to validate the prognostic value of the EMT gene signature. Patients were stratified into different subgroups according to age (≤ 60 versus > 60 years), gender (female versus male), tumor grade (low versus high) and AJCC stage (early-stage versus advanced-stage). The results indicated that high-risk score was significantly associated with a dismal prognosis in young subgroup (HR = 1.810, 95% CI 1.019–3.216, P = 0.043), elder subgroup (HR = 3.298, 95% CI 1.933–5.627, P < 0.001), male subgroup (HR = 3.378, 95% CI 2.057–5.546, P < 0.001), low-grade subgroup (HR = 2.555, 95% CI 1.510–4.325, P < 0.001), high-grade subgroup (HR = 2.218, 95% CI 1.160–4.238, P = 0.016) and early-stage subgroup (HR = 2.402, 95% CI 1.393–4.142, P = 0.002), which suggested that the EMT-related 5-gene signature remained robust predictive capacity across subgroups (Fig. 5).

### Validation of the EMT-related gene signature

**Fig. 6:**
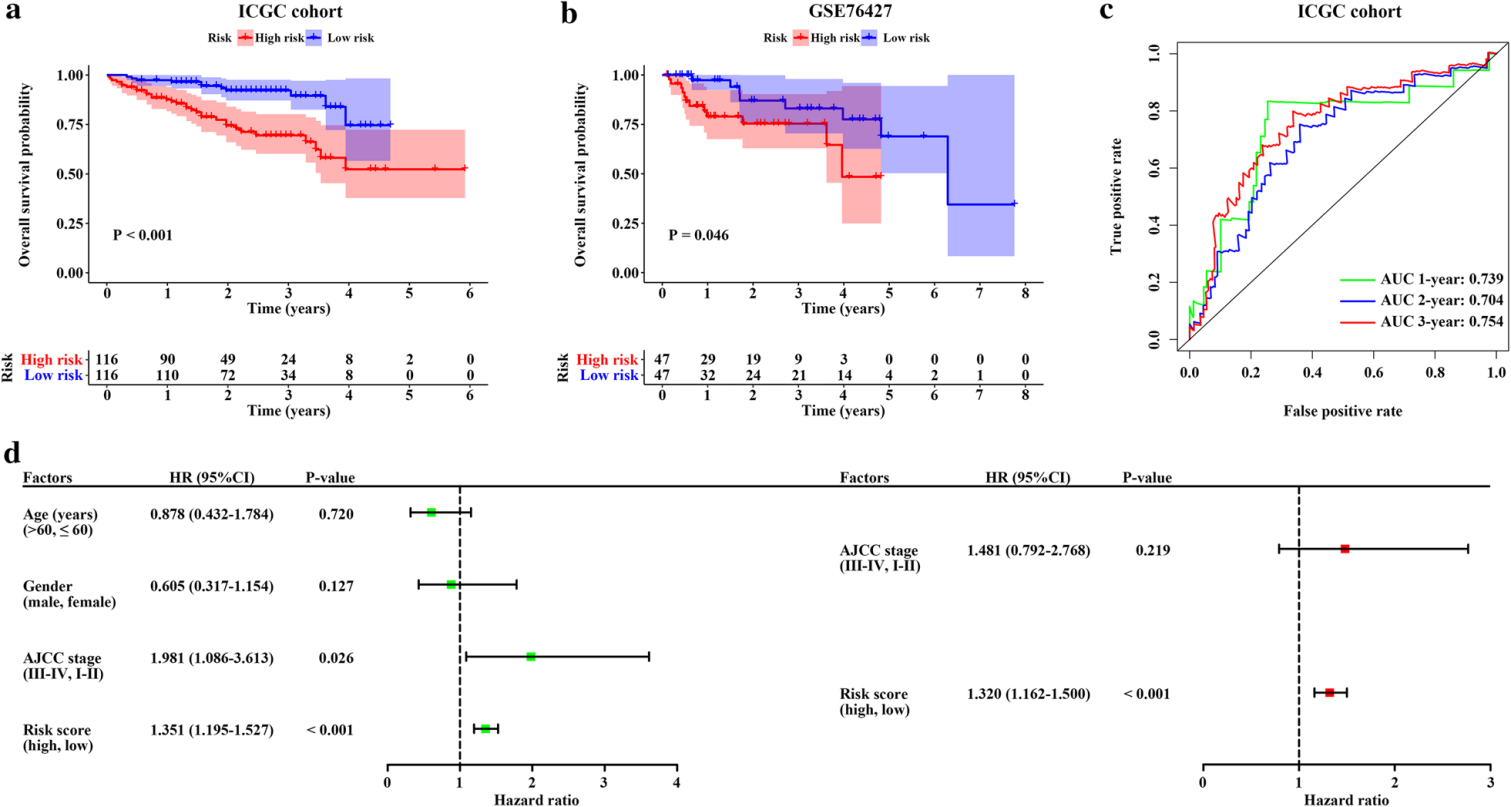
Validation of the 5-gene signature in two external datasets. **a** Kaplan–Meier curve of OS in ICGC cohort. **b** Kaplan–Meier curve of OS in GSE76427 cohort. **c** Time dependent ROC curves for predicting 1-, 2- and 3-year OS of ICGC cohort. **d** Univariate and Multivariate Cox regression analyses of prognostic factors of ICGC cohort

To validate the predictive capacity of the EMT-related 5-gene signature, two datasets (ICGC and GSE76427) were utilized as external validation cohorts. Patients in validation cohorts were also classified into high- and low-risk groups according to median value. In line with the performance in TCGA discovery cohort, we found that patients with high-risk score in ICGC and GSE76427 cohorts had shorter OS than those with low-risk score (HR = 3.340, 95% CI 1.824–6.117, P < 0.001; HR = 2.605, 95% CI 1.015–6.683, P = 0.046) (Fig. 6a, b). In addition, the AUCs for the 1-, 2- and 3-year OS were 0.739, 0.704 and 0.754 in ICGC cohort (Fig. 6c). Similarly, through Cox regression analyses, the EMT-related 5-gene signature could also act as an independent prognostic factor in ICGC cohort (HR = 1.320, 95% CI 1.162–1.500, P < 0.001) (Fig. 6d).

### Establishment and assessment of a predictive nomogram

**Fig. 7:**
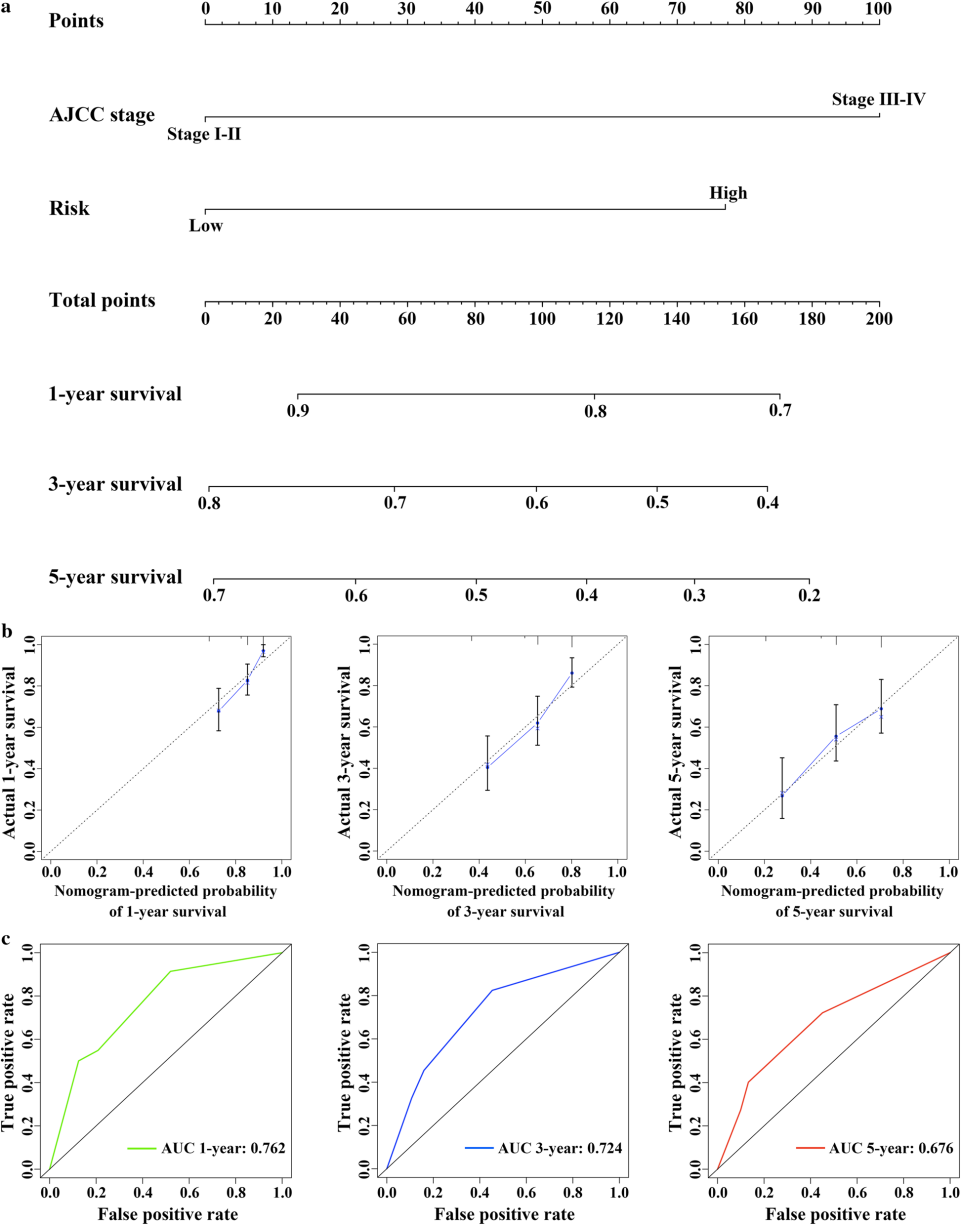
Nomogram construction and evaluation. **a** Nomogram for predicting the 1-, 3- and 5-year OS for HCC patients. **b** Calibration curves for nomogram. **c** Time dependent ROC curves for nomogram

The independent prognostic factors of TCGA discovery cohort, namely the EMT-related 5-gene signature and AJCC stage, were utilized to build a nomogram to provide clinicians with a quantitative method to predict the probability of 1-, 3- and 5-year OS in HCC patients. The point of each factor indicating on the top scaleplate was added up to get a total point which can correspond to the 1-, 3- and 5-year survival rates in the below scaleplates (Fig. 7a). Additionally, calibration plots showed that in comparison to the ideal model, the nomogram presented good performance (Fig. 7b). Besides, the AUCs of nomogram at 1-, 3- and 5-year OS were 0.762, 0.724 and 0.676, respectively (Fig. 7c).

### Identification of EMT-related gene signature associated biological pathways

**Fig. 8:**
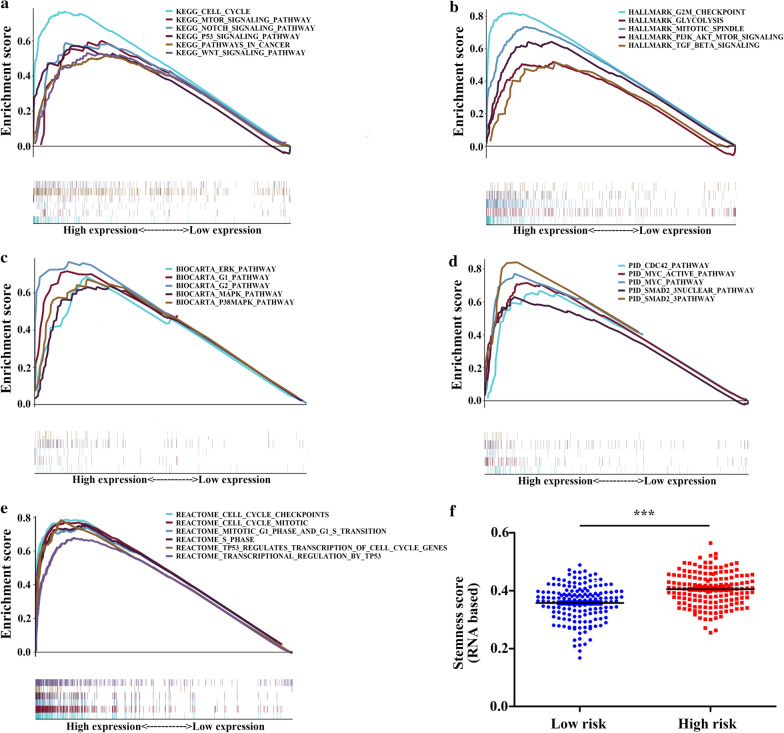
GSEA based on the TCGA cohort to explore the underlying mechanism of the 5-gene signature. Gene set of (**a**) KEGG, (**b**) HALLMARK, (**c**) BIOCARTA, (**d**) PID and (**e**) Reactome associated with the 5-gene signature. Nominal P-value < 0.05 and FDR < 0.25. **f** Association between the 5-gene signature and tumor stemness

GSEA was employed to explore the biological process and signaling pathway underlying the EMT-related 5-gene signature. The result demonstrated that the biological process of cell cycle and glycolysis and several classical cancer-related pathway including PI3K/Akt/mTOR pathway, p53 pathway, Notch pathway, WNT/β-catenin pathway, TGF-β/SMAD pathway and MAPK pathway were significantly enriched in the high-risk group (Fig. 8a–e). Moreover, we found that the high-risk group had a higher stemness score based on mRNA expression which could reflect the tumor stemness than the low-risk group (Fig. 8f).

### Regulatory network and alteration of the 5 hub genes and their expression levels in HCC

**Fig. 9:**
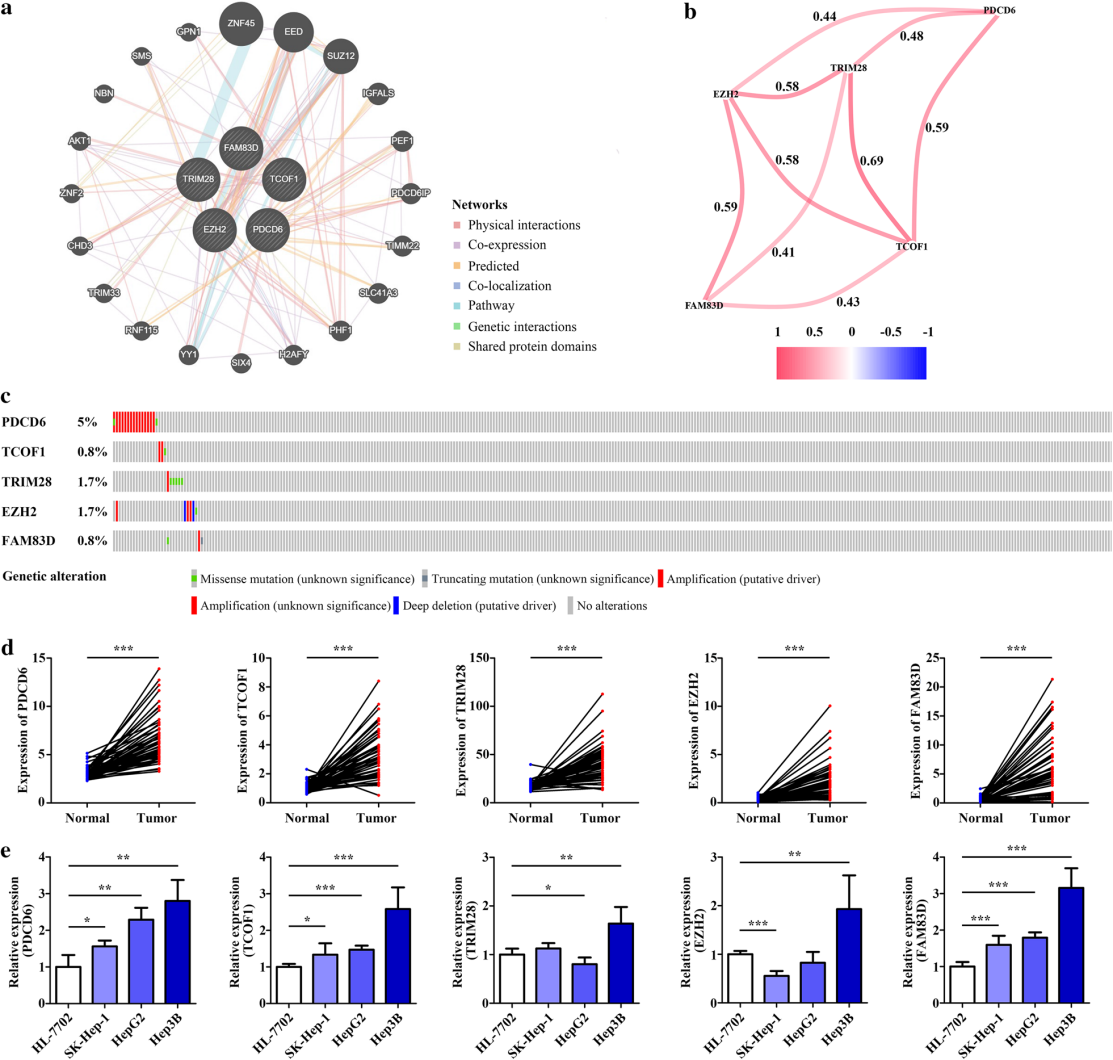
Analysis of the 5 EMT-related genes. **a** GeneMANIA establish a protein–protein interaction network based on these 5 genes. **b** Correlation between the 5 genes in TCGA dataset. **c** The proportion of genetic alteration of the 5 genes in 353 HCC samples from the cBioPortal database. **d** Expression of the 5 genes in the normal samples (n = 50) and paired HCC samples (n = 50) from TCGA dataset. **e** The qRT-PCR analysis of the expression levels of 5 genes in HL-7702, SK-Hep-1, HepG2 and Hep3B cell lines. Data are presented as the mean ± SD, n = 3, *P < 0.05, **P < 0.01 and ***P < 0.001

GeneMANIA was utilized to construct a regulatory network to identify other potential genes that interact with the 5 EMT-related genes. The network contained 5 hub genes and 20 other genes (Fig. 9a), and their connections and weights were listed in Additional file 2. Then, we analyzed the correlation of the 5 hub genes in HCC and found that they were positively correlated with each other (Fig. 9b). The genetic alterations of 5 EMT-related genes were detected via cBioPortal database. The results revealed that the 5 genes were altered in 9% (32/353) of queried samples. Among them, PDCD6 was the most frequently mutated genes (5%), followed by TRIM28 (1.7%), EZH2 (1.7%), TCOF1 (0.8%) and FAM83D (0.8%) (Fig. 9c). In addition, we also investigated the expression levels of 5 EMT-related genes in the normal samples and paired HCC samples based on TCGA cohort. As shown in Fig. 9d, the 5 genes were all highly expressed in the tumor samples. Besides, we employed qRT-PCR to detect their expression levels in normal liver cell line and three HCC cell lines, and found that PDCD6, TCOF1 and FAM83D were highly expressed in three HCC cell lines (Fig. 9e) (Additional file 3).

### Knockdown of PDCD6 inhibits cell migration and invasion in HCC

**Fig. 10:**
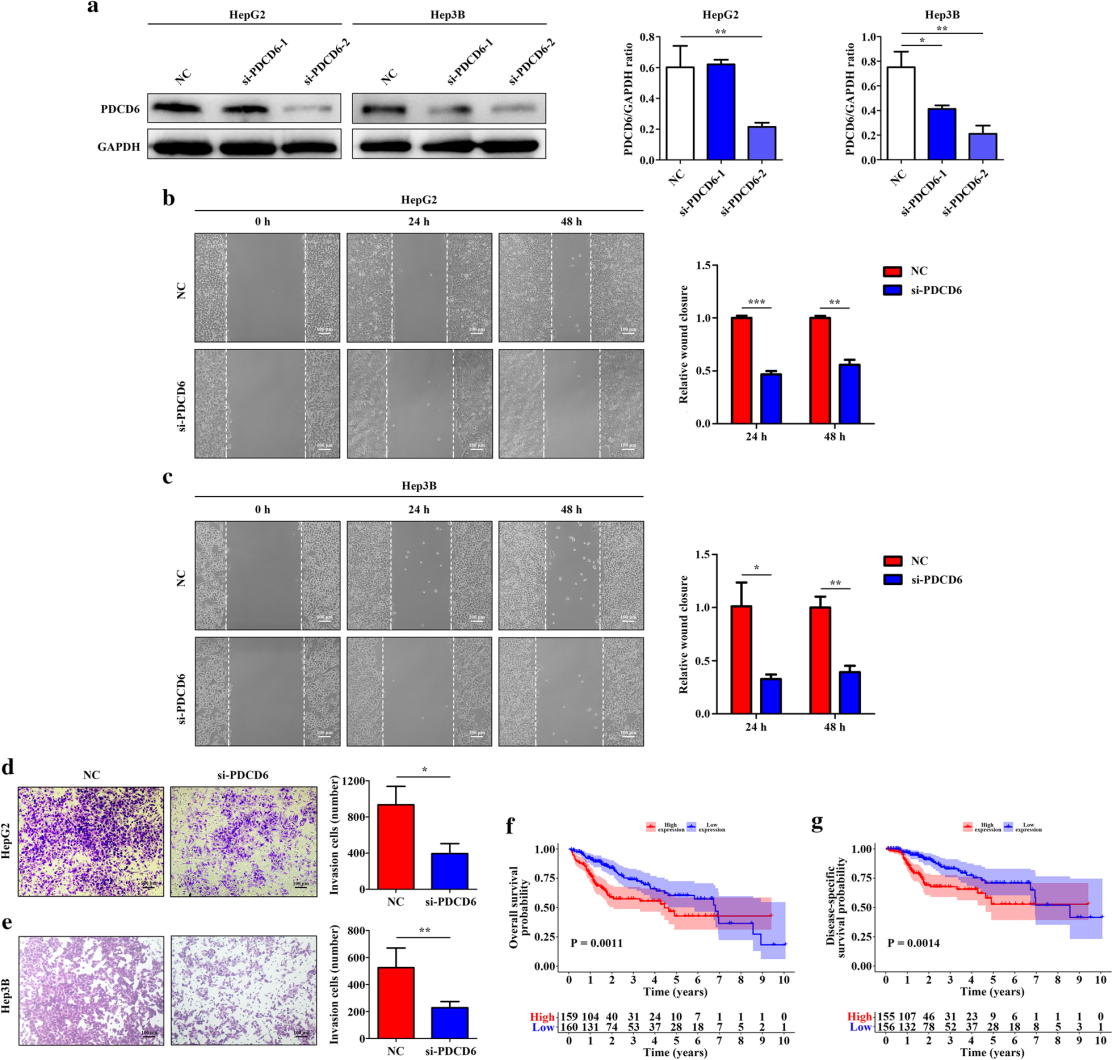
Function of PDCD6 on cell migration and invasion in HCC cells. **a** Knockdown of PDCD6 in HepG2 and Hep3B cells, as determined by western blot. Data are presented as the mean ± SD, n = 3, *P < 0.05 and **P < 0.01. **b, c** Wound healing assay showing cell migration of HCC cells after 24 and 48 h. Data are presented as the mean ± SD, n = 3, *P < 0.05, **P < 0.01 and ***P < 0.001. **d, e** Effects of PDCD6 on cell invasion evaluated by transwell assay. Data are presented as the mean ± SD, n = 3, *P < 0.05 and **P < 0.01. **f** Kaplan–Meier curve of OS between high PDCD6 expression group and low PDCD6 expression group. **g** Kaplan–Meier curve of DSS between high PDCD6 expression group and low PDCD6 expression group

Since the expression of PDCD6 changed most significantly in the three cancer cells and its function in HCC was still unclear, we selected it for further functional exploration. Two different PDCD6 siRNAs and a negative control were transfected into HepG2 and Hep3B cells. Their efficiency in knocking down PDCD6 was detected by western blot. As the result demonstrated, compared to si-PDCD6-1, si-PDCD6-2 performed better and was used for succeeding experiments (Fig. 10a).

The effect of PDCD6 on cell migration was analyzed using wound healing assay. Compared to the NC group, the wound area in PDCD6-knockdown cells was much larger at 24 and 48 h after wounding, which indicated that knockdown of PDCD6 suppressed cell migration (Fig. 10b, c). Moreover, the result of matrigel invasion assay revealed that knockdown of PDCD6 significantly attenuated the invasion capacities of HepG2 and Hep3B cells (Fig. 10d, e). Besides, based on TCGA cohort, HCC patients with high PDCD6 expression had shorter OS (HR = 1.937, 95% CI 1.303–2.879, P = 0.0011) and DSS (HR = 2.262, 95% CI 1.371–3.731, P = 0.0014) than those with low PDCD6 expression (Fig. 10f, g).

## Discussion

HCC is a fatal disease, and patients with HCC have dismal 5-year survival rate. Metastasis and recurrence are the leading reason of the poor prognosis of HCC patients [1]. Previously, EMT has been identified as a prometastatic cellular event that promotes tumor cell invasion and metastasis [15]. Similarly, EMT also plays a critical role in HCC metastasis [11, 16]. Therefore, the EMT-related genes may have great potential to become biomarkers of cancer progression and to predict the prognosis of HCC patients. In this study, an EMT-related 5-gene signature was constructed and presented a favorable performance in predicting the survival of patients with HCC. In addition, we noted that patients in high-risk group were involved in cancer-related pathways such as PI3K/Akt/mTOR pathway and TGF-β/SMAD pathway, which implied a higher risk of progression and metastasis. Moreover, we found that patients with high risk scores were positively associated with tumor stemness, which was in agreement with the conclusion that EMT could make tumor cells have the characteristics of stem cells, and this feature also indicated that patients would have a poor prognosis [10].

The EMT-related 5-gene signature was consisted of TRIM28, EZH2, FAM83D, TCOF1 and PDCD6. TRIM28, a member of the tripartite motif-containing proteins (TRIM) family, is reported to act as an E3 ubiquitin ligase which can form MAGE-C2-TRIM28 and MAGE-A3/6-TRIM28 E3 ligase complexes in cancer to respectively target p53 and AMPK for degradation in a proteasome-dependent way [17, 18]. In HCC, MAGE-C2-TRIM28 complex was found to degrade FBP1, thereby enhancing Warburg effect and promoting cell growth in HCC [19]. EZH2 is a catalytic subunit of polycomb repressive complex 2 (PRC2) and exerts its functions via three kinds of mechanism including PRC2-dependent Lys-27 in histone 3 (H3K27me3) methylation, PRC2-dependent non-histone protein methylation, and PRC2-independent gene transactivation [20]. EZH2 was proved to be closely related to cancer initiation, progression, metastasis, and drug resistance [21, 22]. Previous study found that EHZ2 could be silenced by miR-101, which sensitized HCC cells to chemotherapeutic therapy [23]. FAM83D is found to be overexpressed in various type of cancers such as non-small-lung cancer and colorectal cancer, and also plays a vital role in tumor initiation and progression [24, 25]. Likewise, in HCC, FAM83D promotes cell proliferation and accelerates the G1 to S transition through activating MEK/ERK signaling pathway, which enhances the malignant behavior of tumor [26]. Although the function of TCOF1 is rarely reported in carcinoma, its expression level is high in HCC tissues and HCC cell lines according to our finding, which may provide foundation for the further investigation of the association between TCOF1 and tumorigenesis.

Programmed cell death protein 6 (PDCD6), which is also known as apoptosis-linked gene-2 (ALG-2), was reported to encode a calcium-binding protein and exert its physiological functions via Ca^2+^-dependent interaction with its downstream proteins [27]. PDCD6 has been involved in various physiological processes including signal transduction, cell death and cell division [28]. Meanwhile, PDCD6 is also implicated in the development of cancer, but its role is controversial in different types of tumors. Overexpression of PDCD6 was proved to promote the progression of colorectal cancer via cooperating with C-Raf and activating Raf/MEK/ERK signaling pathway [29]. Similarly, PDCD6 was overexpressed in metastatic ovarian cancer cells and was able to promote cell migration and invasion [30]. Whereas, low expression levels of PDCD6 was found to be associated with a poor prognosis in gastric cancer [31]. In HCC, previous study has shown that PDCD6 is highly expressed in cancerous tissues, but its function is still unclear [32]. Our study found that the expression level of PDCD6 was upregulated in three liver cancer cells compared to normal liver cell. Then, we are the first to explore the function of PDCD6 in HCC, and reveal that knockdown of PDCD6 inhibits cell migration and invasion in HepG2 and Hep3B cells. Furthermore, HCC patients with high PDCD6 expression predicted a dismal prognosis. Therefore, PDCD6 may play a vital role in HCC metastasis and could be an underlying prognostic biomarker and therapeutic target for HCC.

Previous studies have established various gene signatures to predict the prognosis of patients with HCC such as metabolism-related gene signature, glycolysis-related gene signature and so on [33, 34]. Compared with these signatures, the EMT-related 5-gene signature we constructed has higher AUC values for OS, which suggests more excellent performance of our model in predicting prognosis of HCC patients. Nevertheless, our study also has some limitations. Firstly, this prognostic signature is developed using retrospective analysis based on public datasets, thus, the results need to be further confirmed in prospective trails. Secondly, the sample capacity of our validation cohorts is insufficient, especially GSE76427, which affects the accuracy of model prediction to some extent. Finally, the underlying mechanism of PDCD6 promoting HCC cells migration and invasion needs further exploration.

## Conclusion

In summary, we established a novel EMT-related 5-gene signature associated with the prognosis of HCC patients. Validation in two external cohorts (ICGC and GSE76427) further verified the prognostic value of the signature. Moreover, the signature was identified as an independent factor of HCC, and then constructed a nomogram combining with another independent factor to effectively predict the prognosis of HCC patients. Furthermore, knockdown of PDCD6, one of the 5 EMT-related genes, inhibits HCC cells migration and invasion. These results indicate that the EMT-related 5-gene signature can serve as a novel prognostic biomarker for HCC patients and may provide a new mechanism of HCC metastasis.

## Supplementary Information

**Additional file 1: Table S1.** Associations between the clinicopathological characteristics of patients and EMT-related gene signature risk level.

**Additional file 2.** Modes of interaction in PPI network.

**Additional file 3: Figure S1.** Expression of hub genes in cell lines and tissues. **a**. The expression levels of PDCD6 in HL-7702, SK-Hep-1, HepG2 and Hep3B were detected by western blot. Data are presented as the mean ± SD, n=3, *P < 0.05 and **P < 0.01. **b**. The protein expression of TRIM28, EZH2 and TCOF1 in HCC and normal liver tissues. Data were obtained from the Human Protein Atlas (http://www.proteinatlas.org) online database.

## Data Availability

The datasets used and/or analyzed during the current study are available from the corresponding author on reasonable request.

## Abbreviations

EMT: Epithelial-mesenchymal transition
HCC: Hepatocellular carcinoma
TCGA: The Cancer Genome Atlas
ICGC: International Cancer Genome Consortium
GEO: Gene Expression Omnibus
GSEA: Gene set enrichment analysis
ROC: Receiver operating characteristic
qRT-PCR: Quantitative real-time polymerase chain reaction
OS: Overall survival
DSS: Disease-specific survival
AJCC: American Joint Committee on Cancer
NES: Normalized enrichment score
FDR: False discovery rate
cBioPortal: CBio Cancer Genomics Portal
siRNA: Small interfering RNA
NC: Negative control
AUC: Area under curve
HR: Hazard ratio
CI: Confidence interval
PDCD6: Programmed cell death protein 6

## References

[1] Bray F, Ferlay J, Soerjomataram I, Siegel RL, Torre LA, Jemal A (2018). Global cancer statistics 2018: GLOBOCAN estimates of incidence and mortality worldwide for 36 cancers in 185 countries. Cancer J Clin.

[2] Bangaru S, Marrero JA, Singal AG (2020). Review article: new therapeutic interventions for advanced hepatocellular carcinoma. Aliment Pharmacol Ther.

[3] Forner A, Reig M, Bruix J (2018). Hepatocellular carcinoma. Lancet (London, England).

[4] Llovet JM, Montal R, Sia D, Finn RS (2018). Molecular therapies and precision medicine for hepatocellular carcinoma. Nat Rev Clin Oncol.

[5] Diepenbruck M, Christofori G (2016). Epithelial-mesenchymal transition (EMT) and metastasis: yes, no, maybe?. Curr Opin Cell Biol.

[6] Ye X, Weinberg RA (2015). Epithelial-mesenchymal plasticity: a central regulator of cancer progression. Trends Cell Biol.

[7] Huber MA, Kraut N, Beug H (2005). Molecular requirements for epithelial-mesenchymal transition during tumor progression. Curr Opin Cell Biol.

[8] Rhim AD, Mirek ET, Aiello NM, Maitra A, Bailey JM, McAllister F, Reichert M, Beatty GL, Rustgi AK, Vonderheide RH (2012). EMT and dissemination precede pancreatic tumor formation. Cell.

[9] Trusolino L, Bertotti A, Comoglio PM (2010). MET signalling: principles and functions in development, organ regeneration and cancer. Nat Rev Mol Cell Biol.

[10] Mani SA, Guo W, Liao MJ, Eaton EN, Ayyanan A, Zhou AY, Brooks M, Reinhard F, Zhang CC, Shipitsin M (2008). The epithelial-mesenchymal transition generates cells with properties of stem cells. Cell.

[11] Yuan K, Xie K, Lan T, Xu L, Chen X, Li X, Liao M, Li J, Huang J, Zeng Y (2020). TXNDC12 promotes EMT and metastasis of hepatocellular carcinoma cells via activation of β-catenin. Cell Death Differ.

[12] Subramanian A, Tamayo P, Mootha VK, Mukherjee S, Ebert BL, Gillette MA, Paulovich A, Pomeroy SL, Golub TR, Lander ES (2005). Gene set enrichment analysis: a knowledge-based approach for interpreting genome-wide expression profiles. Proc Natl Acad Sci USA.

[13] Warde-Farley D, Donaldson SL, Comes O, Zuberi K, Badrawi R, Chao P, Franz M, Grouios C, Kazi F, Lopes CT (2010). The GeneMANIA prediction server: biological network integration for gene prioritization and predicting gene function. Nucleic Acids Res.

[14] Cerami E, Gao J, Dogrusoz U, Gross BE, Sumer SO, Aksoy BA, Jacobsen A, Byrne CJ, Heuer ML, Larsson E (2012). The cBio cancer genomics portal: an open platform for exploring multidimensional cancer genomics data. Cancer Discov.

[15] Yang J, Weinberg RA (2008). Epithelial-mesenchymal transition: at the crossroads of development and tumor metastasis. Dev Cell.

[16] Ma W, Chen X, Wu X, Li J, Mei C, Jing W, Teng L, Tu H, Jiang X, Wang G (2020). Long noncoding RNA SPRY4-IT1 promotes proliferation and metastasis of hepatocellular carcinoma via mediating TNF signaling pathway. J Cell Physiol.

[17] Doyle JM, Gao J, Wang J, Yang M, Potts PR (2010). MAGE-RING protein complexes comprise a family of E3 ubiquitin ligases. Mol Cell.

[18] Pineda CT, Ramanathan S, Fon Tacer K, Weon JL, Potts MB, Ou YH, White MA, Potts PR (2015). Degradation of AMPK by a cancer-specific ubiquitin ligase. Cell.

[19] Jin X, Pan Y, Wang L, Zhang L, Ravichandran R, Potts PR, Jiang J, Wu H, Huang H (2017). MAGE-TRIM28 complex promotes the Warburg effect and hepatocellular carcinoma progression by targeting FBP1 for degradation. Oncogenesis.

[20] Duan R, Du W, Guo W (2020). EZH2: a novel target for cancer treatment. J Hematol Oncol.

[21] Zingg D, Debbache J, Schaefer SM, Tuncer E, Frommel SC, Cheng P, Arenas-Ramirez N, Haeusel J, Zhang Y, Bonalli M (2015). The epigenetic modifier EZH2 controls melanoma growth and metastasis through silencing of distinct tumour suppressors. Nat Commun.

[22] Sun J, Cai X, Yung MM, Zhou W, Li J, Zhang Y, Li Z, Liu SS, Cheung ANY, Ngan HYS (2019). miR-137 mediates the functional link between c-Myc and EZH2 that regulates cisplatin resistance in ovarian cancer. Oncogene.

[23] Xu L, Beckebaum S, Iacob S, Wu G, Kaiser GM, Radtke A, Liu C, Kabar I, Schmidt HH, Zhang X (2014). MicroRNA-101 inhibits human hepatocellular carcinoma progression through EZH2 downregulation and increased cytostatic drug sensitivity. J Hepatol.

[24] Yu C, Cheng Z, Cui S, Mao X, Li B, Fu Y, Wang H, Jin H, Ye Q, Zhao X (2020). circFOXM1 promotes proliferation of non-small cell lung carcinoma cells by acting as a ceRNA to upregulate FAM83D. J Exp Clin Cancer Res.

[25] Yan L, Yao J, Qiu J (2017). miRNA-495 suppresses proliferation and migration of colorectal cancer cells by targeting FAM83D. Biomed Pharmacother.

[26] Wang D, Han S, Peng R, Wang X, Yang XX, Yang RJ, Jiao CY, Ding D, Ji GW, Li XC (2015). FAM83D activates the MEK/ERK signaling pathway and promotes cell proliferation in hepatocellular carcinoma. Biochem Biophys Res Commun.

[27] Maki M, Takahara T, Shibata H. Multifaceted Roles of ALG-2 in Ca(2+)-Regulated Membrane Trafficking. Int J Mol Sci. 2016, 17(9). 10.3390/ijms17091401 PMC5037681 27571067

[28] Maki M, Suzuki H, Shibata H (2011). Structure and function of ALG-2, a penta-EF-hand calcium-dependent adaptor protein. Science China Life sciences.

[29] Wang X, Wu F, Wang H, Duan X, Huang R, Tuersuntuoheti A, Su L, Yan S, Zhao Y, Lu Y (2020). PDCD6 cooperates with C-Raf to facilitate colorectal cancer progression via Raf/MEK/ERK activation. J Exp Clin Cancer Res.

[30] Su D, Xu H, Feng J, Gao Y, Gu L, Ying L, Katsaros D, Yu H, Xu S, Qi M (2012). PDCD6 is an independent predictor of progression free survival in epithelial ovarian cancer. J Transl Med.

[31] Yamada Y, Arao T, Gotoda T, Taniguchi H, Oda I, Shirao K, Shimada Y, Hamaguchi T, Kato K, Hamano T (2008). Identification of prognostic biomarkers in gastric cancer using endoscopic biopsy samples. Cancer Sci.

[32] la Cour JM, Mollerup J, Winding P, Tarabykina S, Sehested M, Berchtold MW (2003). Up-regulation of ALG-2 in hepatomas and lung cancer tissue. Am J Pathol.

[33] Liu GM, Xie WX, Zhang CY, Xu JW (2020). Identification of a four-gene metabolic signature predicting overall survival for hepatocellular carcinoma. J Cell Physiol.

[34] Jiang L, Zhao L, Bi J, Guan Q, Qi A, Wei Q, He M, Wei M, Zhao L (2019). Glycolysis gene expression profilings screen for prognostic risk signature of hepatocellular carcinoma. Aging.

