# Supplementary material for: An epithelial-mesenchymal transition-related 5-gene signature predicting the prognosis of hepatocellular carcinoma patients
Source: Cancer Cell Int. 2021 Mar 12;21:166. doi: 10.1186/s12935-021-01864-5 (PMC7953549; doi:10.1186/s12935-021-01864-5)
Supplement: Supplementary file 1 — Additional file 1: Table S1. Associations between the clinicopathological characteristics of patients and EMT-related gene signature risk level. [file 12935_2021_1864_MOESM1_ESM.docx]

| **Parameters** | **Number** | **Risk level** | | **P-value** |
| --- | --- | --- | --- | --- |
|  |  | **Low** | **High** |  |
| Age (years) |  |  |  | 0.240 |
| ≤ 60 | 160 | 75 | 85 |  |
| > 60 | 159 | 85 | 74 |  |
| Gender |  |  |  | 0.780 |
| Female | 100 | 49 | 51 |  |
| Male | 219 | 111 | 108 |  |
| Grade |  |  |  | <0.001 |
| G1 | 44 | 30 | 14 |  |
| G2 | 154 | 89 | 65 |  |
| G3 | 109 | 38 | 71 |  |
| G4 | 12 | 3 | 9 |  |
| AJCC stage |  |  |  | <0.001 |
| Stage Ⅰ | 160 | 103 | 57 |  |
| Stage Ⅱ | 76 | 32 | 44 |  |
| Stage Ⅲ | 80 | 22 | 58 |  |
| Stage Ⅳ | 3 | 3 | 0 |  |

**Table. Associations between the clinicopathological characteristics of patients and EMT-related gene signature risk level**

P-values were calculated from χ2 test
