# Supplementary figures and images for: An epithelial-mesenchymal transition-related 5-gene signature predicting the prognosis of hepatocellular carcinoma patients
Source: Cancer Cell Int. 2021 Mar 12;21:166. doi: 10.1186/s12935-021-01864-5 (PMC7953549; doi:10.1186/s12935-021-01864-5)

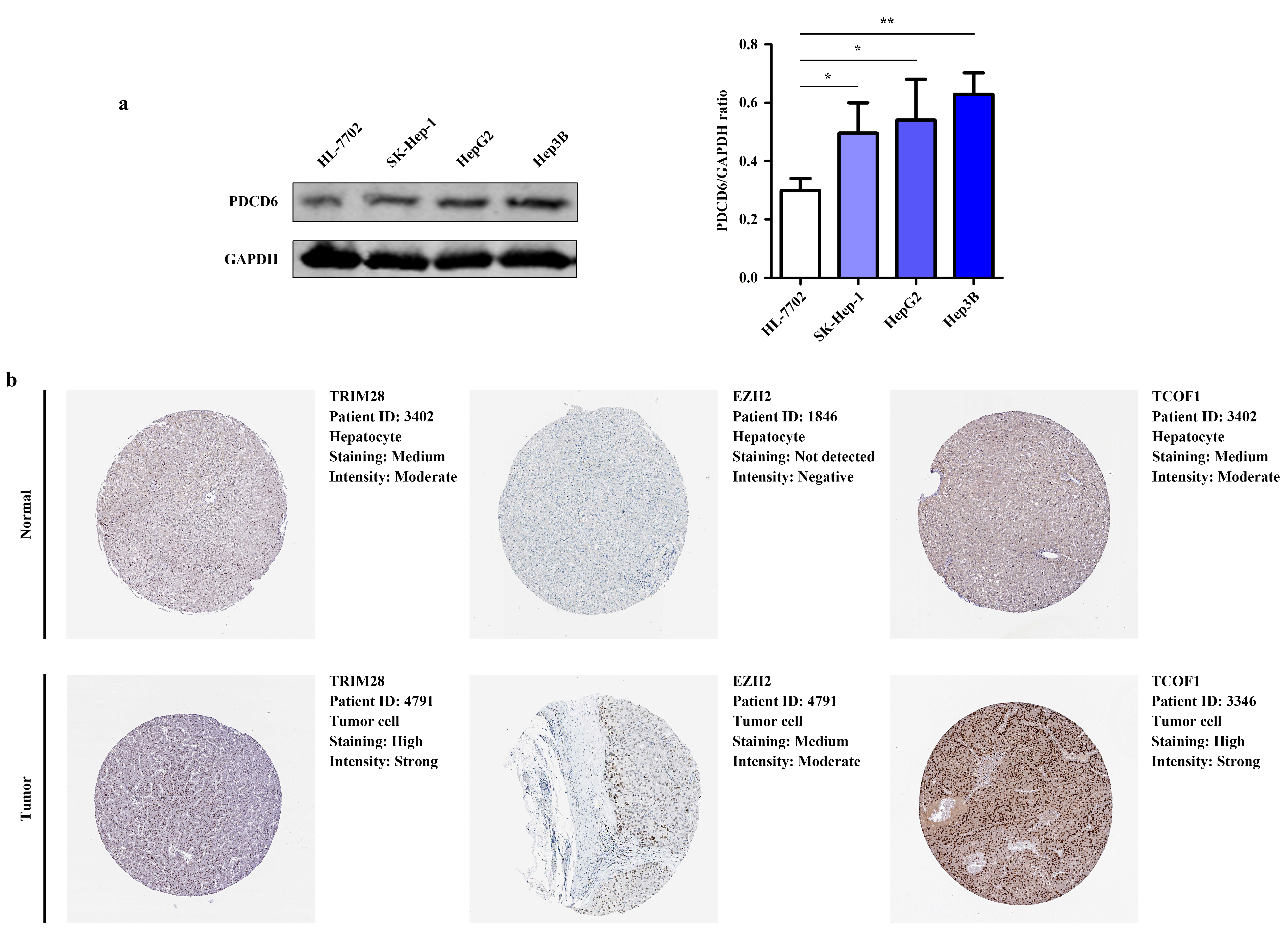

Supplement: Supplementary file 3 — Additional file 3: Figure S1. Expression of hub genes in cell lines and tissues. a. The expression levels of PDCD6 in HL-7702, SK-Hep-1, HepG2 and Hep3B were detected by western blot. Data are presented as the mean ± SD, n=3, *P < 0.05 and **P < 0.01. b. The protein expression of TRIM28, EZH2 and TCOF1 in HCC and normal liver tissues. Data were obtained from the Human Protein Atlas (http://www.proteinatlas.org) online database. [file 12935_2021_1864_MOESM3_ESM.tif]
